# The Green Extraction of Blueberry By-Products: An Evaluation of the Bioactive Potential of the Anthocyanin/Polyphenol Fraction

**DOI:** 10.3390/ijms252011032

**Published:** 2024-10-14

**Authors:** Giorgio Capaldi, Clelia Aimone, Emanuela Calcio Gaudino, Kristina Radošević, Martina Bagović, Giorgio Grillo, Giancarlo Cravotto

**Affiliations:** 1 Department of Drug Science and Technology, University of Turin, Via P. Giuria 9, 10235 Turin, Italy; giorgio.capaldi@unito.it (G.C.); clelia.aimone@unito.it (C.A.); emanuela.calcio@unito.it (E.C.G.); giancarlo.cravotto@unito.it (G.C.); 2 Laboratory for Cell Cultures, Applications and Biotransformations, Department of Biochemical Engineering, Faculty of Food Technology and Biotechnology, University of Zagreb, Pierottojeva Ulica 6, 10000 Zagreb, Croatia; kristina.radosevic@pbf.unizg.hr (K.R.); mbagovic@pbf.hr (M.B.)

**Keywords:** blueberry pomace, microwave-assisted extraction, subcritical water extraction, anthocyanins, polyphenols, bioactivity, green metrics

## Abstract

In the context of a circular economy, this study explores the valorization of blueberry pomace (BP) as a source of bioactive compounds using sustainable extraction methods. Microwave-assisted extraction (MAE) and microwave-assisted subcritical water extraction (MASWE) were employed to obtain two distinct fractions: MAE 1° and MASWE 2°. The first extract, MAE 1°, obtained at 80 °C, had a high total anthocyanin content (21.96 mg_Cya-3-glu_/g_extract_), making it suitable as a natural pigment. Additionally, MAE 1° exhibited significant enzyme inhibition, particularly against α-amylase and β-glucosidase, suggesting potential anti-diabetic and anti-viral applications. The second extract, MASWE 2°, obtained at 150 °C, contained a higher total phenolic content (211.73 mg_GAE_/g_extract_) and demonstrated stronger antioxidant activity. MASWE 2° showed greater inhibition of acetylcholinesterase and tyrosinase, indicating its potential for use in Alzheimer’s treatment, skincare, or as a food preservative. MASWE 2° exhibited cytotoxicity against HeLa cells and effectively mitigated H_2_O_2_-induced oxidative stress in HaCat cells, with MAE 1° showing similar but less pronounced effects. A tested formulation combining MAE 1° and MASWE 2° extracts in a 3:2 ratio effectively enhanced anthocyanin stability, demonstrating its potential as a heat-stable pigment. The extract characteristics were compared with a conventional method (MeOH-HCl in reflux condition), and the protocol’s sustainability was assessed using several green metric tools, which provided insights into its environmental impact and efficiency.

## 1. Introduction

In the context of escalating global environmental concerns and the urgent need for sustainable practices, the valorization of agri-food by-products has become an important focus area [1]. The transition towards more efficient and resilient production chains, as emphasized by the World Resource Institute and the Food and Agriculture Organization, underscores the necessity for long-term strategies that align with ecological sustainability principles [2]. Among the various agri-food by-products, blueberry pomace (BP), a residual matrix from blueberry juice production, represents a significant source of valuable phytochemicals, including dietary fibers, polyphenols, and anthocyanins.

Blueberries (*Vaccinium* spp.), belonging to the Ericaceae family, are small, spherical fruits native to North America [3]. In the past two decades, the global blueberry market has witnessed a remarkable production rate increase of 526%, reaching 1113 M tons in 2021 [4]. Although blueberries are edible as fresh fruit, their seasonal availability and highly perishable nature lead to a significant portion of the crop being utilized for the production of juice, jellies, purees, and other processed products [5]. As a result, BP, comprising seed, skin, and pulp remnants, constitutes a processing by-product accounting for approximately 20–30% of the overall fruit weight in the transformation industry [6]. BP, which can contain more phytochemicals than the whole fruit and the whole plant (i.e., referring to the leaves, trunk, and roots), offers high availability and zero- or even negative-cost starting material for extractables, particularly anthocyanins, making it an excellent matrix for recovering pigments and bioactives [7,8]. Anthocyanins are widely used for their natural coloring properties together with their well-established health benefits [9]. Several studies, including clinical trials and meta-analyses, support the pharmaceutical properties of anthocyanins, such as their anti-inflammatory, antioxidant, anti-hypertensive, anti-hyperglycemic, hypolipidemic, cell-protective, and gut health effects [10,11,12,13]. Therefore, these metabolites are potential natural pharmaceutical ingredients as well as components for nutraceutical and food supplement applications [14]. Among the various approaches to BP valorization—such as producing bio-fuel, bio-char, and bio-oil—the targeted extraction of phytochemicals is currently the most profitable [15]. However, these precious pigments are prone to degradation due to various factors, including pH, light exposure, temperature, co-pigmentation, sulphites, ascorbic acid, oxygen, and enzymatic activity, therefore requiring rigid extraction protocols and storage conditions in order to maintain their integrity [16]. The shift to a circular bio-economy of organic waste necessitates abandoning traditional, resource-intensive extraction methods involving organic solvents and high energy consumption [17]. Environmentally friendly methods like ultrasonic-assisted extraction (UAE), pulsed electric field (PEF), natural deep-eutectic solvent (NaDES), supercritical fluid extraction (SFE), microwave-assisted extraction (MAE), and subcritical water extraction (SWE), as well as the combination of the latter (microwave-assisted subcritical water extraction, MASWE), have shown great potential as valid substitutes to conventional techniques [18,19,20,21,22,23,24].

In particular, MAE exploits electromagnetic irradiation to generate heat using two mechanisms: ionic conduction and dipole rotation. These phenomena lead to a homogeneous heating of the solvent/matrix and help to break down the cell walls of the latter, which promotes the extraction of various compounds. Typical desirable effects are efficient and rapid heating, and other advantages include the use of small devices characterized by low energy consumption and the possibility to work under an inert atmosphere. All of these aspects can lead to improvements in the extraction yield and product quality.

While new green solvents like natural deep eutectic solvents (NaDESs), bio-based solvents, supercritical fluids, and switchable solvents have been widely studied for BP extraction, in this work, we focus on the less-explored method using subcritical water extraction [25,26]. In this context, this study proposes a new extraction protocol for anthocyanins and polyphenols from BP under sequential MAE and MASWE technologies aiming to recover and maintain the integrity of the complete phytochemical profile of BP. Compared to conventional heating methods, microwave (MW) technology provides fast and volumetric heating, leading to the efficient use of energy and improved heat transfer. SWE uses high temperatures (100–374 °C) and pressures to modify water’s properties, decreasing permittivity, viscosity, and surface tension while increasing diffusion and the ionic product concentration. This enhances the solubilization of low-polarity compounds and mass transfer, enabling SWE to mimic organic solvents and extract a wider range of chemical species. The industrial scalability of emerging technologies is a key factor for their potential impact on the industrial market [27]. In this respect, SWE technology has demonstrated a high degree of industrial scalability, indicating its potential for effective valorization in practical market scenarios [28,29].

In this work, to maximize the recovery of anthocyanins and ensure their stability, the first extraction step was optimized by considering the temperature, time, and an acidic media as key parameters. The process design aimed to utilize citric acid as a natural acidifying/stabilizing agent and low-temperature conditions as these factors have been proven to stabilize the chemical structure of anthocyanins and prevent degradation [30]. In the second step, the remaining biomass was subjected to further extraction to recover the less polar and more recalcitrant fractions of polyphenols by applying subcritical water as a solvent.

Both optimized fractions of extracted BP with environmentally friendly methods have been compared with a conventional process to verify any advantages. In these terms, the extracts were tested for their antioxidant activities (chemical and electrochemical methods) and ability to inhibit enzymes (α-amylase, β-glucosidase, acetylcholinesterase, tyrosinase, and elastase), and an in vitro test was carried out on living cells (cytotoxicity, anti-inflammatory, and antimicrobial analyses). In order to enhance the stability of the recovered anthocyanins, the MASWE extract was used as a stabilizer, and thermal degradation was evaluated in different formulations. Finally, the overall sustainability of the designed protocols was evaluated by comparing different green metrics [31,32,33,34,35].

The proposed protocol comprises the above-mentioned sequential steps, which effectively remove target molecules from the matrix. This strategy not only enables the retrieval of anthocyanins and other polyphenolic metabolites but also aims to maintain their bioactivity. In this way, it offers an environmentally friendly alternative to conventional methods that use organic solvents, ensuring the comprehensive valorization of BP’s bioactive profile.

## 2. Results and Discussion

### 2.1. MAE/MASWE Parameter Optimization

**Figure 1:**
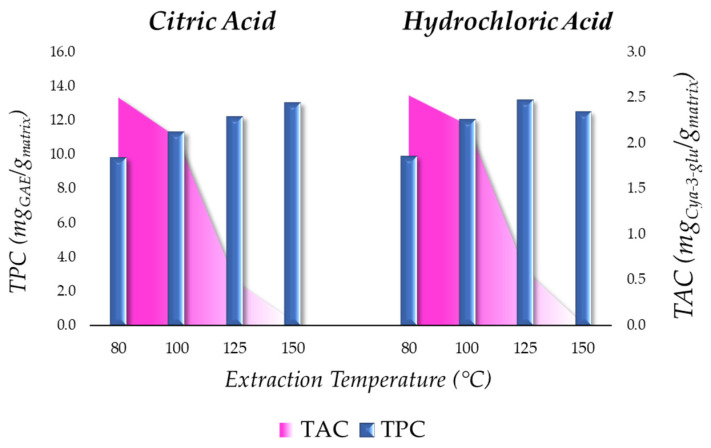
MAE of BP: total polyphenols (mg_GAE_/g_matrix_) and anthocyanin (mg_Cya-3-glu_/g_matrix_) extraction yields at different temperatures using organic and inorganic acid.

BP was subjected to a sequential MAE with the aim of achieving the exhaustive extraction of bioactive compounds. This approach specifically aimed to target the thermolabile species first, and second, the molecules that require harsher conditions to be recovered, thus striving for complete and efficient pomace depletion. The experimental optimization procedure explored the impacts of different extraction temperatures (80, 100, 125, and 150 °C), the use of acidifying agents to stabilize the extracted anthocyanidins (i.e., citric and hydrochloric acid), and in a second step, the extraction time (Figure 1). Non-acidified water extraction was not considered due to stability issues, as reported in the literature [16].

Consistent with the well-documented study on anthocyanin degradation, the temperature increase showed to denature TAC in the extract to a value of 0 when reaching 150 °C [36]. Interestingly, the level of TPC showed a reverse trend with respect to anthocyanins. This indicates that while anthocyanins were degrading, other classes of polyphenols were being consistently extracted, with the highest yields being obtained at 150 °C (13.06 and 12.53 mg_GAE_/g_matrix_ for citric acid and hydrochloric acid, respectively). This outcome evidences the advantage of a double extraction strategy in order to save pigments from degradation (at lower temperature) and efficiently retrieve the remaining polyphenols still present in the pomace in a second step (at a higher temperature).

The two different acid stabilizers adopted did not appear to significantly influence the stability of anthocyanins during extraction (Figure 1). The highest yields, obtained at 80 °C, were 2.50 and 2.52 mg_Cya-3-glu_/g_matrix_, along with extract selectivity values of 21.96 and 24.93 mg_Cya-3-glu_/g_extract_ with citric acid and hydrochloric acid, respectively. Considering factors beyond the simple yield, the selection of the most suitable acid was carefully evaluated. Firstly, the use of hydrochloric acid requires an additional removal step due to its potential health hazards, making it expensive in terms of time and energy, presenting major operational risks when also accounting for the threat of residues in the final product. Secondly, it was reported that solvents containing hydrochloric acid in the extraction process may lead to the degradation of pigments during concentration [37]. Taking these aspects into account, citric acid appears as a more favorable choice than hydrochloric acid, even when considering sustainability and renewable feedstock exploitation, with this additive being bio-derivable.

**Figure 2:**
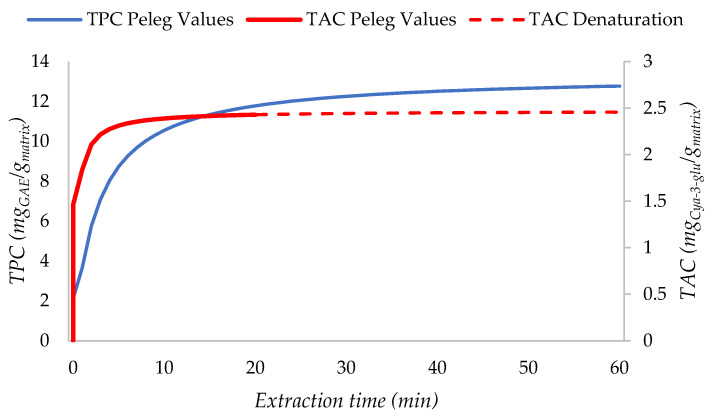
The Peleg regression model applied for the optimization of the MAE time for polyphenols and anthocyanin in a citric acid solution. The experimental input parameters were time (2, 5, 10, 20, 30, 45, and 60 min), temperature (80 °C), and S/L (1:30).

After fixing those parameters for MAE (80 °C, S/L: 1:30, citric acid), we investigated the extraction kinetic to minimize time and energy consumption while maximizing the TAC by applying the Peleg regression model [38]. Different extraction times (ranging from 0 to 60 min) were evaluated under the optimal investigated temperature (80 °C) to determine their impact on the TPC and TAC (Figure 2).

The extraction trend expressed by the Peleg model shows two different extraction kinetics for anthocyanin and total polyphenols. The two linearization equations were

TAC linearization equation y = 0.4048x + 0.1388 R^2^ = 0.9989 (1)

TPC linearization equation y = 0.075x + 0.1798 R^2^ = 0.9815 (2)

The anthocyanin plateau, representing the maximum extraction yield, was reached at 20 min. During this initial timeframe, the extract’s selectivity toward anthocyanin increased constantly, reaching its peak at 21.97 mg_Cya-3-glu_/g_extract_. Extended extraction led to anthocyanin degradation, with selectivity decreasing to 20.7, 19.4, and 19.3 mg_Cya-3-glu_/g_extract_ at 30, 45, and 60 min, respectively. The Peleg regression model does not take into consideration the degradation phenomena. The curve’s steady-state effect is justified by the simultaneous coexistence of the degradation of anthocyanins and increase in the total extraction yield, which balances out the losses and stabilizes the average anthocyanin yield. Considering energy and time consumption, along with higher extract selectivity, the optimal MAE time for BP anthocyanin recovery is 20 min.

In contrast, the yield of polyphenols continues to increase until the 60 min mark, displaying a different extraction kinetic at 80 °C compared to anthocyanins, with the optimal total yield occurring between 45 and 60 min. However, if the primary goal of extraction is the retrieval of anthocyanins, it is crucial to prioritize their efficient extraction within the optimal timeframe of 20 min. Non-anthocyanin polyphenols, which remain in the residual BP biomass, can be effectively recovered in subsequent extraction steps, thereby preventing the wastage of bioactive compounds.

In summary, the first extraction step assisted by MW technology was set at 80 °C for 20 min with citric acid and resulted in a TAC of 21.97 mg_Cya-3-glu_/g_extract_, achieving a total yield of 2.50 mg_Cya-3-glu_/g_matrix_. Additionally, the TPC selectivity and yield reached 86.44 mg_GAE_/g_extract_ and 9.83 mg_GAE_/g_matrix_, respectively.

Typically, anthocyanins need to be extracted using an organic solvent, such as methanol or ethanol, often with a controlled pH. In fact, when these solvents (either pure or mixed with water in varying percentages) and a controlled pH are used, the total anthocyanin recovery is generally higher compared to water extraction, and the total yield can be nearly twice with ethanol than water [39].

The ineffectiveness of using water as an election solvent is also due to the thermal sensitivity of this class of compounds, making it impossible to reach the subcritical state of water, which could otherwise enhance recovery. However, the aim of this work was to leverage microwaves and water to maximize the extraction of anthocyanins present in the matrix. The optimized result is higher than that using other green extraction methods, such as PEF-assisted extraction, even when using organic solvents (2.50 mg_Cya-3-glu_/g_matrix_ vs. 1.76 mg_Cya-3-glu_/g_matrix_) [40], while it is consistent with the results obtained by Lee et al. using water at 80 °C (2.3 mg vs. 2.50 mg_Cya-3-glu_/g_matrix_) [41]. The extraction conditions in these aforementioned trials were optimized to maximize the anthocyanin content, with the optimal temperature being 80 °C, and under these conditions, polyphenol recovery was comparable with the polyphenol recovery of MAE 1°, namely 7.38 mg_GAE_/g_matrix_ vs. the 9.83 mg_GAE_/g_matrix_ value obtained using our system. Since the polyphenols remaining in the biomass after anthocyanin recovery are not as thermosensitive, it was possible to proceed with a sequential polyphenol extraction step, harnessing the properties of subcritical water, especially the changes in polarity, thus leading to further valorization of the biomass.

**Figure A1:**
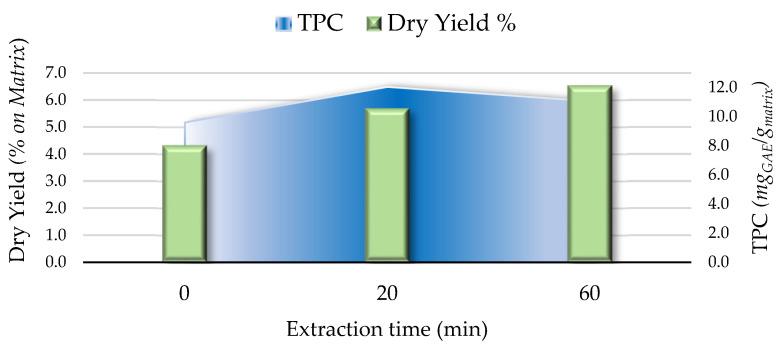
Polyphenol time optimization extraction at 150 °C in MASWE 2°.

**Figure A2:**
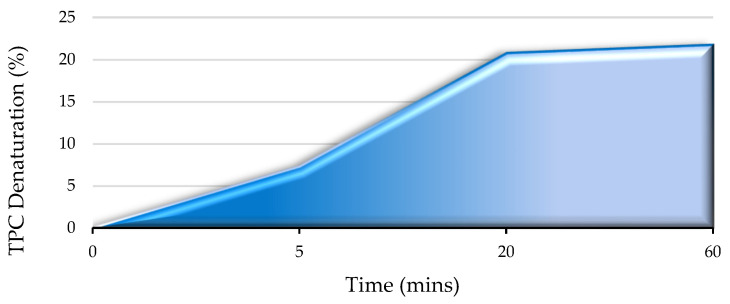
Degradation kinetic of polyphenols in MASWE 2° at different times at 150 °C.

Moving forward to step two, according to the results shown in Figure 1, the highest polyphenol extraction efficiency in BP was achieved at 150 °C. Consequently, the second extraction phase was conducted at this temperature, investigating the process kinetic (0, 5, 20, and 60 min) using simple water as a solvent, since stabilizing anthocyanins was no longer necessary. Increasing the extraction time enhanced the total yield but resulted in a lower level of polyphenol extraction (Figure A1). To evaluate the potential polyphenol degradation during the process, the extract recovered at 0 min was subjected to 150 °C for 5, 20, and 60 min. The results indicate that degradation increased within the initial 20 min, with approximately a 20% decrease, followed by stabilization (Figure A2). By matching this trend with the extraction outcomes, it is possible to state that metabolite recovery dominates in degradation in the first 20 min (against high degradation, we still observe a TPC increase). On the contrary, at 60 min, even if the degradation percentage is very low (*approx*. 1% compared to 20 min), a contraction in the TPC can be observed. Thus, it is possible to conclude that at that time, extraction is no longer able to counterbalance the degradation phenomena, even if limited. These considerations led us to define the optimal duration for the second protocol (at 150 °C) in 20 min, yielding a value of 12.05 mg_GAE_/g_matrix_ with an extract selectivity of 211.73 mg_GAE_/g_extract_. Regarding the total polyphenol extraction, when comparing MASWE with another green technology, such as PEF-assisted extraction, the results are of a comparable order of magnitude (10.52 mg_GAE_/g_matrix_ vs. 12.05 83 mg_GAE_/g_matrix_, respectively, for PEF-assisted extraction and MASWE 2°) [40]. However, in this study, an ethanol-based solvent is used, which poses a limitation in extraction processes, particularly when considering the work from a perspective of industrial applicability and scalability, due to its high cost, safety concerns, and the regulatory requirements it must comply with, especially when compared to the ultimate green solvent, water, which offers excellent performance under subcritical conditions. The results obtained using this technology demonstrate an improvement compared to other studies in the literature that use only water as a solvent, as seen in the aforementioned result of 7.38 mg_GAE_/g_matrix_, whereas a recovery of 12.05 mg GAE/g DM was achieved using subcritical water, representing an average improvement of 30% [41].

### 2.2. Laboratory Scale-Up and Extract Characterization

The optimized small-scale extraction conditions obtained for BP in a sequential approach were reproduced by scaling up the protocol from 1 g to 25 g and exploiting the same MW system. The resulting extracts were then compared with a hydroalcoholic protocol, which served as the benchmark extraction (Table A1). The laboratory scale-up protocol did not show major changes in the extraction performance.

**Figure 3:**
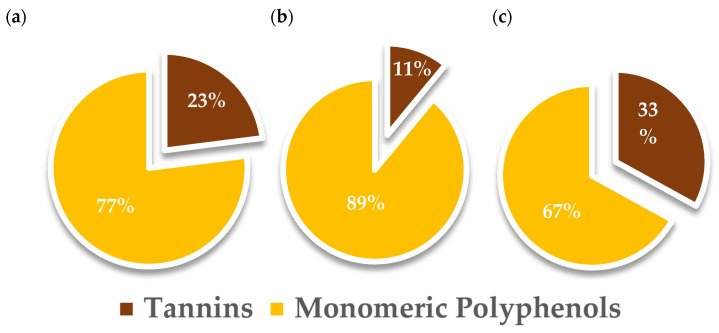
Monomeric polyphenols and tannins’ relative percentages regarding total polyphenol content in BP extracts. (**a**) MAE 1°; (**b**) MASWE 2°; (**c**) Conv.

The conventional benchmark showed a higher yield of TPC (23.46 mg_GAE_/g_matrix_) with respect to both MW extracts (10.44 and 7.20 mg_GAE_/g_matrix_ for MAE and MASWE, respectively). The distribution of polyphenols between the monomeric and tannin fractions was evaluated (see Figure 3), revealing a predominance of the monomeric fraction in both water solvent extracts (84.32 and 134.39 mg_GAE_/g_extract_ for MAE and MASWE, respectively) compared to the hydroalcoholic solvent extract (77.58 mg_GAE_/g_extract_).

The higher presence of monomeric phenolic compounds in MAE and MASWE samples may lead to beneficial implications in terms of the nutraceutical value as polyphenols with major polymerization degrees tend to have lower intestinal absorption rates and, as a consequence, reduced bioavailability [42].

On other hand, the retrieval of TAC reached with the green extraction protocol did not reach the level obtained in the conventional protocol (2.11 vs. 3.61 mg_Cyan-3-glu_/g_matrix_ for MAE and Conv., respectively).

However, it is worth noting that both MW-assisted protocols achieved the above results without the aid of a hydroalcoholic solution and HCl in one-sixth of the time (20 min vs. 120 min). In detail, *approx.* 75% and 60% of the overall TPC (mg_GAE_/g_matrix_) and TAC (mg_Cyan-3-glu_/g_matrix_), respectively, were recovered through the cascade approach. It is important to state that this preliminary test helps to point out the feasibility of a future industrial scale-up as it starts to evaluate whether the proposed process could suffer from any peculiar shortcoming against transposition, including but not limited to a mixing problem, reproducibility issues, and product degradation due to process harshness.

### 2.3. Antioxidant Activity

**Figure 4:**
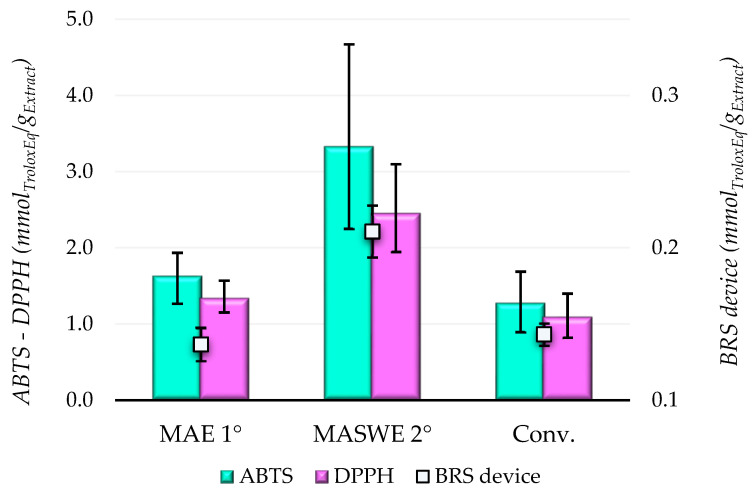
Antioxidant activity of BP extracts measured with chemical (ABTS; DPPH) and electrochemical methodologies (BRS device).

Blueberry extracts were subjected to two tests, namely DPPH· and ABTS·+ assays, to directly evaluate their scavenging activity on free radicals through the hydrogen atom transfer (HAT) and single-electron transfer (SET) mechanisms [43,44]. The antioxidant capacity of the extracts was also measured in terms of electrochemical transmission (based on voltammetry, amperometry, and coulometry) with the BRS device. By employing these different methodologies, a comprehensive evaluation of the antioxidant potential of BP extracts was achieved (Figure 4).

The antioxidant activity measured with the DPPH· and ABTS·+ tests revealed a correlated trend with the monomeric polyphenol fraction of the extracts. Remarkably, both microwave-assisted BP extracts demonstrated significantly higher TEAC values compared to the conventional extract. Specifically, the DPPH· values were 1.342 and 2.419 mmol Trolox Eq./g extract, and the ABTS values were 1.633 and 3.335 mmol Trolox Eq./g extract for MAE and MASWE, respectively. This is notable even though the conventional extract had a higher TPC, with DPPH· and ABTS values of 1.096 and 1.277 mmol Trolox Eq./g extract, respectively. Hence, it is possible to state that the monomeric polyphenolic fraction presents major antioxidant features compared to the polymeric one. The electrochemical antioxidant activity quantification confirmed the overall results of the colorimetric assays, demonstrating higher repeatability with a lower relative error compared to conventional chemical analytical methods. Indeed, the highest conductivity value was showed by MASWE 2° (0.197 mmol_TroloxEq_./g_extract_), followed by Conv. (0.146 mmol_TroloxEq_./g_extract_) and MAE 1° (0.142 mmol_TroloxEq_./g_extract_). Even if in this case, the Conv. extract showed a slightly higher value than MAE 1°, the results are not statistically different.

### 2.4. Bioactivity Performance

#### 2.4.1. Enzyme Inhibition Activity

Blueberry extracts have demonstrated significant potential in treating various human diseases due to their rich phytochemical content. These natural compounds are known for their antioxidant, anti-inflammatory, and enzyme inhibitory properties, which contribute to their therapeutic benefits [45]. To comprehensively explore the multifaceted potential applications of BP extracts across different fields, a series of in vitro enzymatic inhibition assays were conducted. In particular, these assays were designed to target enzymes linked to common diseases such as diabetes (α-amylase) and neurodegenerative disorders (acetylcholinesterase, AChE). Furthermore, the assays evaluated the antiproliferative effects (β-glucosidase) and anti-melanogenesis properties (tyrosinase) of the extracts. Additionally, the potential use of those extracts as anti-browning agents for vegetable preservation was investigated through the inhibition of tyrosinase (Table 1).

α-Amylase is a pancreatic enzyme responsible for catalyzing the hydrolysis of starch in a smaller unit, which is then further hydrolyzed by α-glucosidase to a glucose unit [42]. These two enzymes combined increase blood glucose levels and postprandial hyperglycemia, posing a significant problem for individuals with diabetes mellitus. Therefore, inhibiting these enzymes could help mitigate the metabolic disorders associated with this condition. The EC50 value for the BP extract inhibition of α-amylase was determined only for the MAE 1° extract, which was 0.0213 mmol_AE_/g_extract_. The conventional sample exhibited a high color matrix signal that, together with low activity, made readings impossible up to a concentration of 5.57 mg/mL, where inhibition was around 35%. Conversely, the MASWE 2° extract, which showed a less pronounced matrix effect, was tested at concentrations of up to 30 mg/mL without any inhibition feature. These findings align with the study conducted by Xiaodan Hui et al., which demonstrated the α-amylase inhibitory activity of blueberries and attributed this effect to their anthocyanin content [46].

β-Glucosidase is an enzyme involved in the breakage of the β-1,4-glycosidic bond and is most associated with the hydrolysis of cellobiose to glucose and in the processing of glycoproteins for the development of an outer envelope of virus cells [47]. Due to the double effect of this enzyme class, its inhibition is potentially connected to the treatment of type 2 diabetes, and it can also be used as an anti-viral agent [48]. Even for this test, the most pronounced inhibitor effect was shown by MAE 1°, followed by Conv. and MASWE 2°.

Acetylcholinesterase is a serine enzyme involved in the hydrolysis of acetylcholine, an important neurotransmitter, into acetate and choline. Reduced levels of these neurotransmitters have been noted in patients with Alzheimer’s disease; thus, the inhibition of the enzyme responsible for neurotransmitter cleavage is studied as an enhancer of treatments for neurodegenerative disorders [49]. On the other hand, common AChE inhibitors, such as carbamates and organophosphate, can lead to severe toxicity and can be used as natural pesticides [50]. In nature, some inhibitors of AChE with major chances have been identified to be implemented in pharmaceutical formulations for Alzheimer’s treatment. In this study, the BP extract showed an activity similar to AchE inhibitors, with MASWE 2° having the highest efficacy, followed by MAE 1° and Conv.

Tyrosinase (also known as polyphenol oxidase) is a copper-based enzyme involved in the hyperpigmentation or melanogenesis of human skin and in the enzymatic browning of fruit and vegetables [51]. Inhibitors of this enzyme can be applied in both the cosmetic field as anti-browning skincare products and in the food industry as preservatives. The inhibition potential of BP extracts was particularly high in MASWE 2°, followed by MAE 1° (Table 1). As reported for α-amylase, the matrix color effect of the conventional sample, together with low activity, made the detection of EC50 impossible, allowing us to detect a maximum inhibition of 25% at a concentration of 9.86 mg/mL.

In summary, the MAE 1° BP extract demonstrated a higher inhibitory effect on α-amylase and β-glucosidase, while the MASWE 2° BP extract exhibited greater inhibition of acetylcholinesterase and tyrosinase. These findings suggest that the two extracts could be applied in different fields, thereby expanding their market opportunities in parallel directions. Specifically, MASWE 2° verified the preliminary results regarding its antioxidant activity, indicating its potential as a natural preservative. However, it is important to note that, despite some observed activities, the inhibition potential of these extracts was significantly lower compared to standard inhibitory compounds. Future research should focus on developing techniques to identify and isolate the specific molecules responsible for the observed enzymatic inhibition activities. Purification toward the targeted inhibitory active compound would enhance the efficacy of BP extracts for designed applications.

#### 2.4.2. Antiproliferative Activity

**Figure 5:**
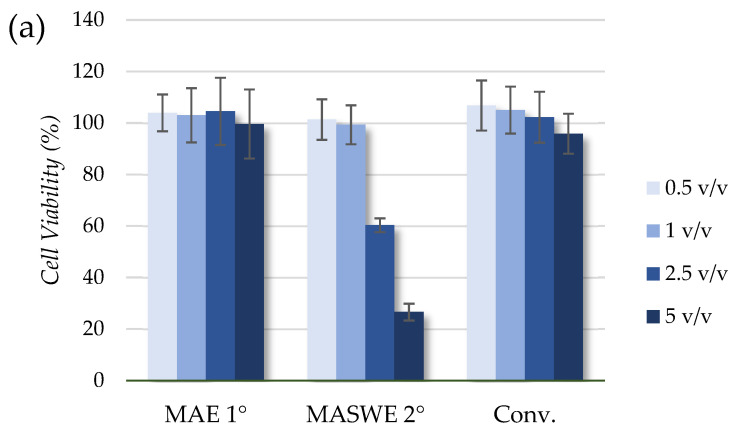

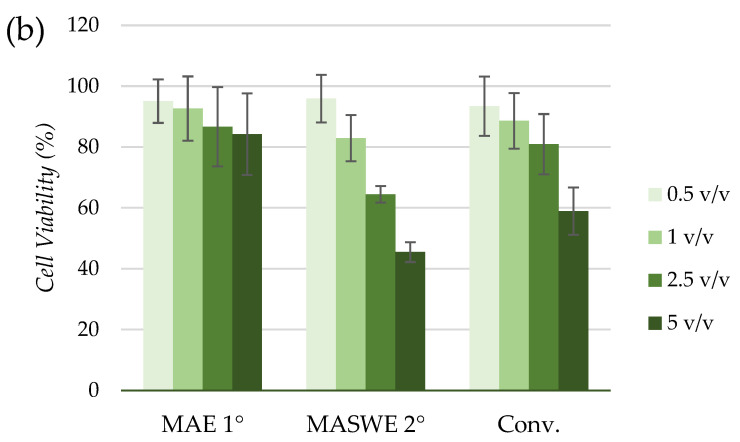
Effects of different volume ratios (*v*/*v*) of three tested extracts (MAE 1°, MASWE 2°, and Conv.) on proliferation of HeLa (**a**) and HaCaT (**b**) cells. Results are expressed as cell viability (%) relative to control cells.

Blueberry phytochemicals are often assessed in vitro on different cell lines, whereby the treatment of cells often includes a whole extract, delivered as a reconstituted powder, juice, or pomace [52]. Herein, the antiproliferative activity of three extracts (MAE 1°, MASWE 2°, and Conv.) was assessed by examining their effects on the growth of HeLa tumor cells and healthy HaCaT cells. The antiproliferative activity of the prepared samples was determined, and the results are expressed as the cell viability percentage of treated cells compared to control (non-treated) cells, as illustrated in Figure 5a,b.

All three tested extracts displayed a similar effect toward HeLa and HaCaT cells, meaning that the volume ratios (0.5 and 1 *v*/*v*) did not have significant antiproliferative activity toward HeLa and HaCaT cells. When the cells were treated with the higher volume ratios (2.5 and 5 *v*/*v*) of the MASWE 2° and Conv. extracts, a more pronounced effect on cell growth was observed, with the lowest detected cell viability of 26.60 ± 3.26% in HeLa cells treated with 5 *v*/*v* of MASWE 2°. Such results are not completely correlated with the determined TPC values, which are the highest for Conv., followed by MASWE 2° and then MAE 1° (Table A1). On the other hand, there is a correlation with the presence of the monomeric fraction in the extracts and the antiproliferative effects, which are the highest for MASWE 2° (Figure 3).

#### 2.4.3. Antioxidative Activity in Cells with Induced Oxidative Stress

**Figure 6:**
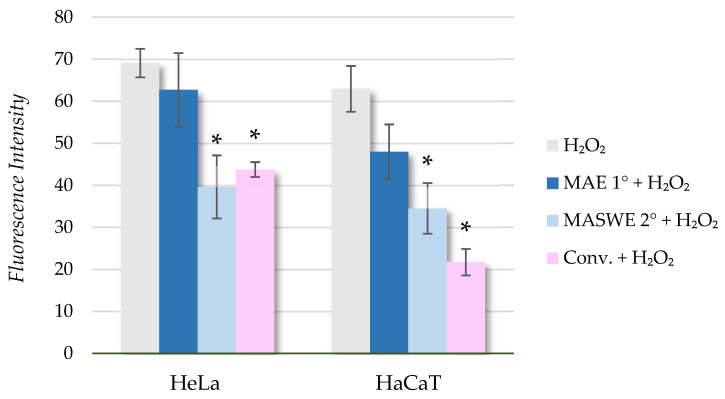
Measurement of spectrophotometric fluorescence intensity. Results are expressed as mean ± SD. Asterisks represent significant difference in reactive oxygen species (ROS) content determined by DCF-DA assay between positive control (H_2_O_2_) and treatment groups (* *p* < 0.05).

The antioxidant effects of blueberry extracts or blueberry phytochemicals are reported in numerous studies. These studies have demonstrated that treatment with those compounds can attenuate oxidative stress in various cell types, primarily by reducing the formation of ROS. Additionally, blueberry extracts have been shown to enhance scavenging activity and decrease lipid peroxidation [53]. The antioxidant activity of the obtained fractions was determined by a DPPH· assay, showing that the highest potential is found in MASWE 2°, followed by Conv. and MAE 1° (Figure 4). Therefore, we wanted to determine the potential of these extracts to protect HeLa and HaCaT cells from oxidative stress induced by the addition of H_2_O_2_. The cells were pretreated with 1.0 (*v*/*v*) for a certain time, and then the DCF-DA assay was used as a simple and cost-effective way to detect the total cellular ROS. DCF-DA is a permeable dye that crosses the cell membrane and, in the presence of ROS, is oxidized by cellular esterases into a fluorescent form (DCF), which can be detected spectrofluorometrically. When an antioxidant is added to a cell culture, it can scavenge peroxyl radicals, thereby reducing DCF formation. The results obtained by the DCF-DA assay are presented in Figure 6.

When comparing the formation of ROS (induced by H_2_O_2_) in HeLa and HaCaT cells pretreated with extracts (1.0 *v*/*v*) with positive control, it is evident that MASWE 2° and Conv. have a protective effect on induced oxidative stress in both HeLa and HaCaT cells. A statistically significant effect of BP extract pretreatment on the decrease in % ROS was achieved by the addition of the MASWE 2° and Conv. extracts, whereas MAE 1° did not show a protective effect even though it also had a relatively high DPPH· value. The protective impact on HaCaT cells is slightly more pronounced than that in HeLa cells, although the trend is similar in both cell lines, with no significant difference between tumor and healthy cells.

The cell culture-based models and assays, like those used here, are not sufficient to draw definitive conclusions about the effects of BP compounds and/or extracts on complex physiological processes in the human body. But, despite their limitations, such studies are valuable, and they consistently demonstrate that blueberry components possess antioxidant properties, which contribute to the overall health and functional benefits associated with blueberries.

### 2.5. Thermal Stability

The thermal stability of targeted molecules is a crucial value in the food industry, considering the necessity of microorganism inactivation with thermal procedures. These protocols use a temperature ranging from 65 °C (low-temperature long-time pasteurization, LTLT) to 121 °C for sterilization procedures. The commercialization of pigment or bioactive ingredients that can be subjected to a high temperature with minimal losses is a current challenge. In these terms, the BP extracts were tested under accelerated thermal degradation at different temperatures to assess the kinetic constants, the activation energy, and the pre-exponential factor of the different anthocyanin in BP extract formulations. The tested samples were MAE 1° and Conv., along with a control sample without the use of citric acid (Contr.) as a stabilizer and a formulation obtained by combining the MAE 1° extract with the MASWE 2° extract in a ratio of 3:2 (1° + 2°). This last experiment was performed to assess the possible application of MASWE 2° as a preserving and thermostabilizing agent due to its high bioactive potential, as elucidated through its antioxidant activity (Section 2.3) and tyrosinase inhibition (Section 2.4.1).

Among the three different reaction kinetic linearization models tested, the first-order reaction kinetics demonstrated the best fit, with *R*^2^ ≥ 0.95 for all of the degradation patterns examined (Table A2 and Table A3). As a consequence, the slopes derived from the degradation kinetics were treated as first-order rate degradation constants (k = −q). These constants showed an absolute value increase proportional to the rise in temperature, indicating accelerated degradation at higher temperatures. These constants were then used to calculate the activation energy (AE) for the degradation of anthocyanin by linearizing the equation in the function of the first-order rate degradation constant at a given temperature (T). Equation (3) reports the linearization of the Arrhenius equation for the k value (T is expressed in Kelvin and R = 8.3143 J K^−1^ mol^−1^).

(3) $$\ln\left(k\right)=\frac{AE}{RT}+A$$

**Figure 7:**
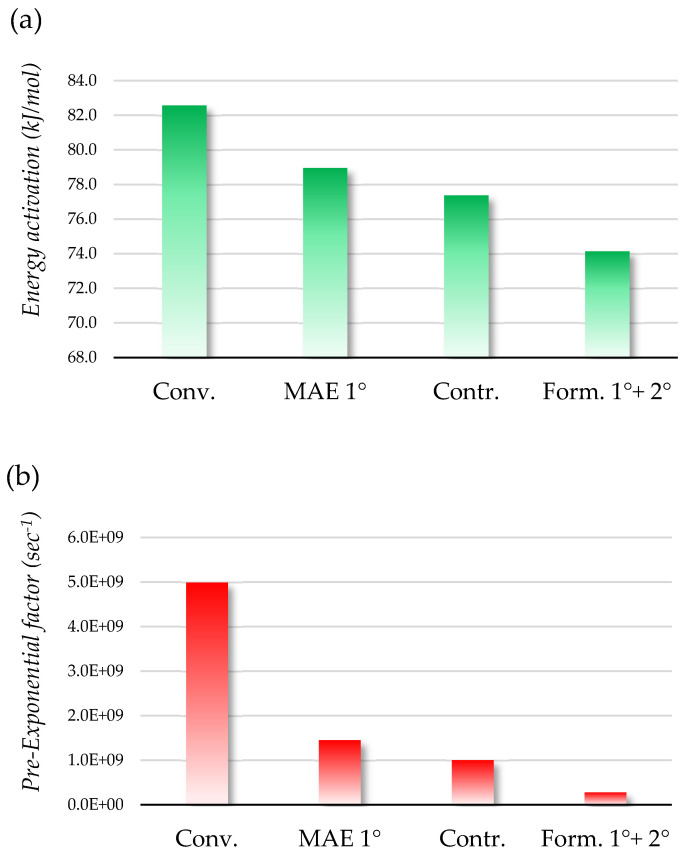
Energy activation (**a**) and pre-exponential factor (**b**) for tested BP formulation. Green bars: factor considered directly proportional to thermal stability; red bars: factor considered inversely proportional to thermal stability.

This equation describes the relationship between the reaction rate constant and temperature. AE represents the energy required to initiate a chemical reaction and serves as a metric for characterizing the stability of anthocyanin at various temperatures. On the other hand, the intercept of the equation, which is the pre-exponential factor (A), represents the frequency of collisions and the orientation of reactant molecules. Generally, higher AE values indicate greater stability and consequently lower degradation rates, while a lower pre-exponential factor suggests a reduced likelihood of the reaction occurring. The AE and A for the four tested formulations showed to be independent from each other, and so the total stability must consider both factors to be reliable (Figure 7a,b).

**Figure 8:**
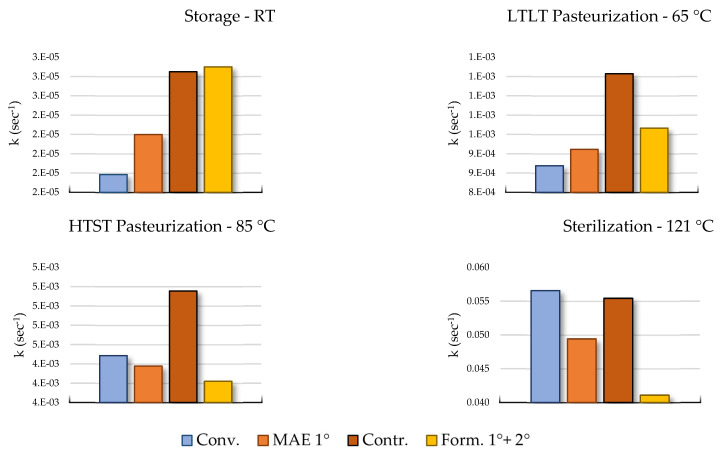
The four graph bars report the degradation constant (k) calculated for the four key temperatures in food processing. The degradation constant value is inversely proportional with thermal stability at the relative temperature.

In these terms, the EA and A factors were used to calculate the kinetic constant of degradation at different temperatures, reflecting different thermal processes applied in the food chain (storage at RT, 25 °C), low-temperature long-term pasteurization (LTLT, 65 °C), high-temperature short-term pasteurization (HTST, 85 °C), and sterilization (121 °C). The comparisons are reported in Figure 8.

The use of citric acid as a preservative confirmed its efficacy with MAE 1°, showing a lower degradation constant for each extrapolated process temperature. On other hand, as expressed by the degradation constant, the relative stability of the other three formulations varies as the temperature changes. Formulations such as Conv. exhibit the highest resistance at lower temperatures like the storage temperature and LTLT, followed by MAE 1°, Contr., and Form. 1° + 2°. In contrast, at higher temperatures (typical of HTST and sterilization processes), the Form. 1° + 2° and MAE 1° samples show the lowest degradation constants and thus the highest anthocyanin stability. These findings suggest that water extracts are less prone to degradation at high temperatures compared to hydroalcoholic extracts, making them more suitable for thermal stabilization procedures. Furthermore, the results indicate that MASWE 2° could potentially serve as a preservative to mitigate anthocyanin degradation during high-temperature processing procedures, confirming the activity previously reported.

### 2.6. A Sustainability Assessment of the Process

Shifting to more sustainable production models is an urgent challenge in current large-scale production. While frameworks such as the Sustainable Development Goals (SDGs) and the twelve principles of green chemistry provide valuable guidelines, they currently lack specific criteria to quantify and standardize the environmental performance on extractions [54,55]. These criteria are essential to accurately assess the sustainability of new protocols to effectively evaluate the impact of innovative processes.

**Figure 9:**
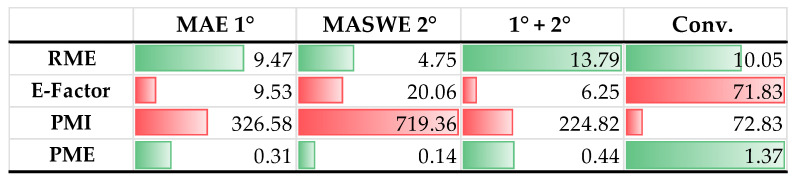
Green metrics evaluation for BP extraction procedures. Green bars: metrics considered directly proportional to sustainability; red bars: metrics considered inversely proportional to sustainability.

In the chemical industry, this assessment is facilitated by the use of so-called “green metrics”. Green metrics evaluate both the environmental impact and protocols’ efficiency, considering factors such as energy consumption, performance, yield, and operator safety. In these terms, the current work evaluated the sustainability level of the MAE 1° and MASWE 2° extracts described for BP and the whole cascade approach, adopting the hydroalcoholic benchmark protocol as a reference. Firstly, these methods were screened using the most exploited metrics gained from green chemistry, namely RME, E-Factor, PMI, and PME (Figure 9), and they were later screened with more recent and improved parameters [30].

The RME value indicates the level of depletion of biomass and, subsequently, the amount of produced waste. In this case, even if the conventional benchmark has major values with respect to the non-traditional technique, the combined process allows for a major recovery of bioactives from the BP, thus producing a lower volume of waste. However, the RME does not consider solvents and their involvement in waste generation. In this scenario, the E-Factor allows for the inclusion of solvent in the calculation, providing a more comprehensive analysis. Thus, the use of ethanol and hydrochloric acid intensively affected the evaluation, showing outcomes that were 7.5-, 3.5-, and 11.5-fold worse with respect to MAE 1°, MASWE 2°, and the combined process. However, the E-Factor has the huge bias of not considering water in the computation. Therefore, the use of PMI or the more intuitive PME is recommended for extraction processes. With these metrics, the conventional process performed better because in the protocol, the solid/liquid ratio was 1:5 *w*/*v* compared to the 1:30 *w*/*v* used for the alternative techniques.

**Figure 10:**
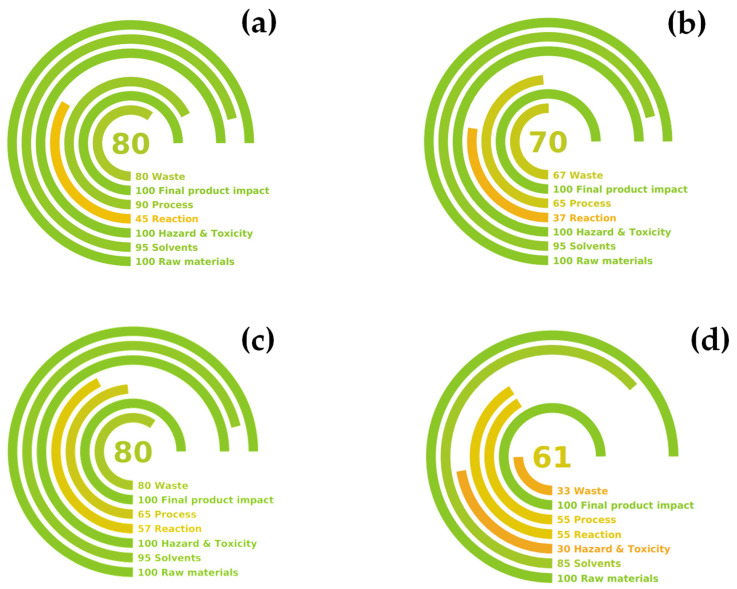
Green Motion evaluation scores for BP extractions. (**a**) MAE 1°, (**b**) MASWE 2°, (**c**) 1° + 2°, and (**d**) Conv.

This first four examples of possible green metrics are just the tip of the iceberg for a more comprehensive evaluation of the sustainability concept. Indeed, these examples only consider yield and waste production as sustainability factors, excluding important aspects such as procedure hazardousness, compound toxicity, energy consumption, involved equipment, and product quality. When taking these aspects into consideration, more holistic green metrics appeared recently, such as the Green Certificate, AgrePrep, Complex GAPI, and Green Motion [31,32,33,34]. The use of these metrics is most commonly associated with green analytical techniques rather than industrial extraction procedures, but some aspects can be borrowed and applied in this field. Table A4 reports the application of recent green metrics for the extraction procedure, highlighting the strengths and weaknesses of each technique. Considering the overall performance and the potential application, the Green Motion approach was chosen as the most suitable one for the evaluation of the BP extraction procedure. Hence, Figure 10 shows a comparison between the earned Green Motion values.

The Green Motion metric is divided into seven main criteria. The raw material score was assessed to be 100 in all tested procedures since we used biomass as raw material, so the renewable carbon percentage was complete. When using water as a solvent, the protocol lost 5 points on a scale of 100, whilst the use of the hydroalcoholic mixture in the conventional process resulted in a value of 15 on a scale of 100. Meanwhile, HCl addition was considered in the “*Hazard & Toxicity*” evaluation, losing 70 points out of 100. The “*Reaction*” step was the main agent in the overall score for the MW-assisted approach as a consequence of pressurization. In terms of the “*Process*” score, the non-conventional system consumed more energy (MAE 1° 796 Wh, MASWE 2° 1192 Wh and Conv. 524 Wh), but this fact is counterbalanced by the extraction rate (20 min vs. 120 min of the Conv.); thus, the overall quantification shifted in favor of MAE 1° and MASWE 2°.

The “*Final Product Impact*” criterion was assessed as completely sustainable for all three processes. Finally, the “*Waste*” value was calculated through the application of the E-Factor, which, as assessed before, supports the MW-assisted strategy (see Figure 9). The final Green Motion output resulted in scores of 80 and 70 for MAE 1° and MASWE 2°, respectively, while the conventional procedure obtained 61 points. If we take into consideration the complete valorization strategy for BP, the integrated protocol (1° + 2°) earns a total score of 80. This result reflects the synergy between MAE 1° (faster and more energy-efficient) and MASWE 2° (more energy-expensive but able to enhance biomass depletion).

*Green Motion* is a valuable tool for calculating the sustainability of extraction procedures. However, it has limitations such as neglecting critical aspects like energy consumption quantification, the heating process nature, the carbon footprint analysis, and the use of the simple *E-Factor* for waste evaluation.

These gaps emphasize the need for more advanced and comprehensive sustainability assessment methods in the field of green extraction. Future research should aim to develop frameworks that address these shortcomings to provide a more thorough understanding of environmental impacts associated with extraction techniques.

## 3. Materials and Methods

### 3.1. Blueberry Pomace and Chemicals

The BP was provided by Indena Spa (Settala, Milano, Italy) as industrial residue from juice extraction (*Vaccinium myrtillus* L.). The matrix was stored at −20 °C in dark conditions to prevent degradation and subsequently utilized in frozen form. The moisture content as well as the organic/inorganic ratios were determined by means of thermogravimetry using a muffle furnace (Nabertherm GmbH, Lilienthal, Germany). The experimental protocol involved dehydration at 100 °C for 24 h, followed by calcination at 650 °C for 4 h. Thermogravimetric analysis on BP had the following results: water content of 67.17%; organic fraction of 32.69%; and inorganic fraction of 0.12%. All solvents and reagents utilized in this study were purchased from Sigma-Aldrich (St. Louis, MO, USA).

### 3.2. Microwave and Microwave-Assisted Subcritical Water Extraction

**Figure 11:**
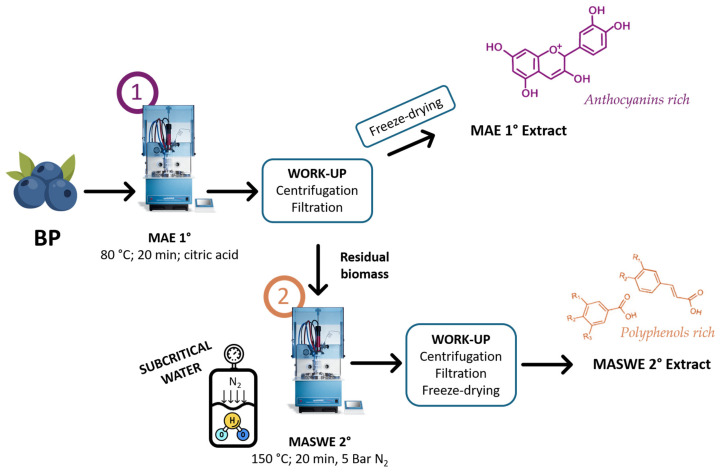
Schematic representation of processing operation for MAE (1) and MASWE (2) extract production.

The extractions were performed in a SynthWAVE multimodal reactor (Milestone, Bergamo, Italy) able to exploit external inert gas feeding (N_2_). Each experiment involved three repetitions of N_2_ purging to eliminate any residual oxygen, which could cause oxidative stress on the biomass. Finally, N_2_ was loaded up to 5 bar to avoid ebullition. Distilled water was used as the extraction solvent, and an acidifying agent was added to achieve pH 3 and prevent degradation of anthocyanins. Two acidifying agents were tested: hydrochloric acid (46 µg/mL) and citric acid (0.5 mg/mL). The optimization protocol for extraction first involved investigating the impacts of temperature and acidifying agent and then the extraction time. Four temperatures, ranging from 80 to 150 °C, were tested for each acidifying agent for 20 min. Other parameters such as biomass loading (1 g), solid-to-liquid (S/L) ratio (1:30), maximum irradiation power (1500 W), and magnetic stirring (650 rpm) were maintained throughout the investigations, and other parameters were not specified. The resulting solution was filtered under a vacuum, and the biomass was thoroughly washed with fresh water. The dry extract was recovered by lyophilization (LyoQuest-85, Tel-star, Madrid, Spain), weighed, and stored at 4 °C for further analyses. The extracted samples were evaluated for dry yields, total phenolic content, and anthocyanin content. After optimizing the extraction temperature, a kinetic study was performed at 80 °C using citric acid as the acidifying agent for the following times: 0, 2, 5, 10, 20, 30, 45, and 60 min. The time of “0” corresponds solely to the heating ramp. The optimized sample (MAE, 80 °C for 20 min) was then subjected to a cascade extraction approach at 150 °C, and three extraction times were investigated: 0, 20, and 60 min. The optimized multi-step protocol was then reproduced on a larger scale, using 25 g (the extracts derived from the optimized first and second extractions were denominated MAE 1° and MASWE 2°, respectively). In Figure 11, a schematic representation of the MAE and MASWE protocols is presented.

### 3.3. General Procedure for Conventional Extraction

A benchmark reference for the total polyphenol and anthocyanin contents was provided by conventional extraction in a hydroalcoholic solution (EtOH:H_2_O ratio of 60:30) in the presence of hydrochloric acid (0.8% *v*/*v*_H_2_O_) [56]. The sample (10 g) was subjected to extraction using 225 mL of hydroalcoholic solution (S/L 1:5) under reflux conditions and magnetic stirring (650 rpm) in a silicon oil bath maintained at 55 °C for 120 min. The resulting extract was filtered, and the ethanol was eliminated by means of a rotavapor operating at 175 mbar and 40 °C before being freeze-dried. The resulting product was used as a benchmark reference and labeled as Conv.

### 3.4. Colorimetric Assay

#### 3.4.1. Total Polyphenol Content (TPC) Determination

The total polyphenol content (TPC) was determined by the Folin–Ciocalteau assay [57]. A calibration curve of gallic acid in water solution with dilutions ranging from 5 to 250 µg/mL was used as a reference to determine the polyphenol content in the BP extracts. Each test tube contained 250 µL of sample dissolved in deionized water, followed by the sequential addition of 4 mL of distilled water, 500 µL of Na_2_CO_3_ solution (10% *w*/*v*), and 250 µL of Folin–Ciocalteau reagent. The solution was shaken and stored at room temperature in dark conditions for 25 min. After incubation, absorbance was measured using a UV-vis spectrophotometer (Cary 60, Agilent Technologies, Santa Clara, CA, USA) at 725 nm in a plastic cuvette (1 cm). TPC was expressed as mg/g of gallic acid equivalents (GAEs) in terms of selectivity and yield. The TPC selectivity is expressed as mg_GAE_/g_extract_, whilst the TPC yield is expressed as mg_GAE_/g_matrix_. The considered values are always reported on dry matter. All measurements were conducted in triplicate.

#### 3.4.2. Total Anthocyanin Content (TAC) Determination

The total anthocyanin content (TAC) was evaluated using the pH differential method [58]. Two solutions were prepared by mixing 1 mL of the BP extract dissolved in water with 5 mL of potassium chloride buffer (0.02 5 M KCl, pH 1 with HCl) and sodium acetate buffer (0.4 M CH_3_CO_2_Na, pH 4.5 with HCl). After allowing the solutions to equilibrate for five minutes, the absorbance was measured at 510 nm and 700 nm, with deionized water being used as a blank.

The summary absorbance of the samples was calculated as shown in Equation (4):

(4) $$A={\left(A_{\lambda \;vis-max}-A_{700}\right)}^{pH1.0}-{\left(A_{\lambda \;vis-max}-A_{700}\right)}^{pH4.5}$$

The anthocyanin content in the samples was calculated using Equation (5):

(5) $$Anthocyanin\;content=\frac{A\times MW\times DF}{\varepsilon \times 1}$$

MW represents the molecular weight, and ε is the molar absorptivity of anthocyanin pigment in the acidic aqueous solvent. The MW and ε values used in this formula correspond to cyanidin-3-glucoside, where MW = 449.2 and ε = 26.900. All measurements were conducted in triplicate.

#### 3.4.3. Total Tannin Content Determination

Tannin determination was conducted using a modified Peri and Pompei protocol [59]. Briefly, 600 µL of a hemisulfate cinchonine solution (0.5% *w*/*v*) was added to 600 µL of the BP extract solution in a 1.5 mL Eppendorf tube. The mixture was shaken and incubated overnight at 4 °C to facilitate the precipitation of cinchonine complexed tannins. The sample was then centrifuged at 26,000 rpm for 2 min at 10 °C (Allegra 64R, Beckman Coulter Srl., Roma, Italy). The resulting supernatant was separated from the precipitate and analyzed using the Folin–Ciocalteau assay. The total tannin content was calculated using the difference between the initial sample and the analyzed supernatant post-precipitation and expressed as gallic acid equivalents (GAEs).

#### 3.4.4. Antioxidant Activity

##### DPPH· Assay

The radical scavenging activity of the extracts was evaluated using the stable free radical 2,2-diphenyl-1-picrylhydrazyl (DPPH·) according to the method described by Brand-Williams et al. [60]. The inhibition of DPPH· was monitored by measuring the decolorization of the solution and compared to a Trolox methanolic solution. Using serial dilutions, solutions in methanol with different concentrations of the dry extracts were prepared starting from an initial concentration of 0.5 mg/mL, and absorbance was measured at 515 nm. Absorbance data were processed using Bobo Least Squares software (ver. 0.9.1) to generate a Probit regression [61]. The scavenging activity was expressed as an EC50 value, determined as the extract concentration inhibiting 50% of the DPPH· radical, and Trolox Equivalent Antioxidant Capacity (TEAC). A blank sample (only methanol) and a reference sample (without DPPH·) were used to normalize the results and evaluate the matrix effect. All measurements were conducted in triplicate.

##### ABTS· + Assay

A modified procedure based on Re et al. was used to determinate the antioxidant activity toward ABTS·+ salt (2,2′-azino-bis acid(3-ethyllbenzothiazolin-6-sulfonic) [62]. A solution with 38.40 mg of ABTS·+ and 6.6 mg of K_2_S_2_O_8_ in 10 mL of water was incubated overnight and then diluted through a 1:100 ratio with methanol. For the test, 500 µL of stock solution of the extract (at decreasing concentrations) and 500 µL of ABTS·+ MeOH reagent were combined and incubated at 30 °C for 6 min. Absorbance at 734 nm was measured, and inhibition percentages were calculated and processed by Probit regression with the Bobo Least Squares software [60]. The antioxidant activity was expressed as EC50 mg/mL and TEAC. A blank water solution and a reference sample (without ABTS·+) were used to normalize results and evaluate the matrix effect. All measurements were conducted in triplicate.

##### Electrochemical Antioxidant Activity Determination

The antioxidant activity was quantified using the BRS device (BQC Redox Technologies, Oviedo Asturias, Spain), an electrochemical instrument specifically designed for the rapid, precise, and straightforward assessment of the redox state in diverse samples [63]. The BP extracts were solubilized in distilled water and subsequently diluted in a 1:1 ratio with the provided electrolyte solution. Each assay utilized a BRS disposable strip, with 50 µL of the solution being applied to the test area. Measurements are expressed in microcoulombs (µC) and were converted to Trolox equivalents via a calibration curve integrated within the instrument.

#### 3.4.5. Enzyme Inhibition Activity

##### α-Amylase Inhibition Test

α-Amylase inhibition was evaluated using a standardized protocol with minor modifications [64]. Briefly, the sample preparation involved mixing 100 μL of the extract (concentration range: 1–10 mg/mL) with 200 μL of pH 6.9 phosphate-buffered saline (PBS) buffer and 100 μL of α-amylase solution (1.22 mg/mL in PBS) in a glass vial. The mixture was then incubated at 25 °C for 20 min. Subsequently, 200 μL of substrate solution (5 mg/mL starch) was added, and the mixture was further incubated at 25 °C for 10 min. The reaction was terminated by adding 1 mL of 3,5-dinitrosalicylic acid (DNS) solution and incubating the mixture at 100 °C for 5 min. After cooling, the reaction mixture was diluted with 8 mL of distilled water. The absorbance of the resulting solution was measured at 540 nm. Standard acarbose was used as a standard inhibitor, and controls were performed without the inhibitor (reference) and without starch for every extract dilution (blank).

##### β-Glucosidase Inhibition Test

β-Glucosidase inhibition activity was assessed using the following protocol with minor modifications [64]. Samples (1–10 mg/mL) were mixed with PBS buffer (0.1 M, pH 5) and β-glucosidase solution (0.005 mg/mL in PBS). After a 10 min incubation period at 37 °C, the reaction was initiated by adding substrate solution (1.5 mg/mL *p*-nitrophenyl-β-D-glucopyranoside) and further incubation at 37 °C for 10 min. The reaction was terminated by adding 700 μL of Na_2_CO_3_ (pH 10). Absorbance was measured at 410 nm. Castanospermine served as the standard inhibitor, and controls included samples without inhibitor (reference) and without enzyme (blank).

##### Acetylcholinesterase Inhibition Test

The acetylcholinesterase (AChE) inhibitory activity of the extracts was evaluated using a modified Ellman’s method [65]. In an Eppendorf tube, 160 μL of the extract was mixed with 520 μL of PBS (0.1 M, pH 8.0). To this mixture, 60 μL of acetylcholinesterase solution (0.05 U/mL in PBS, pH 8.0) and 100 μL of 5,5′-dithiobis-(2-nitrobenzoic acid) (DTNB, 0.25 mg/mL in PBS, pH 8.0) were added. The reaction mixture was incubated at 37 °C for 30 min. Subsequently, 160 μL of acetylthiocholine iodide substrate solution (0.25 mg/mL in PBS) was added, followed by another 30 min incubation period at 37 °C. Absorbance was measured at 405 nm. Donepezil was used as the standard inhibitor. A reference reaction without any inhibitor and a blank sample without the enzyme’s substrate for each extract dilution were included. The percentage inhibition of AChE activity was calculated by comparing the absorbance readings of the samples, references, and blanks.

##### Tyrosinase Inhibition Test

Following the methodology described by Di Petrillo et al. (2016), the tyrosinase inhibition activity of BP extracts was assessed [66]. A reaction mixture was prepared by combining 50 μL of the extract with 50 μL of tyrosinase solution (2500 U/mL) and 850 μL of PBS buffer (0.1 M; pH 6.8). This mixture was incubated at 37 °C for 20 min. Subsequently, 50 μL of L-tyrosine (1.5 mg/mL) was added, and the mixture was incubated again at 37 °C for 20 min. Absorbance was measured at 492 nm. Kojic acid was used as the standard, and a control without the inhibitor was included to establish baseline activity. Blanks, without L-tyrosine, were prepared for each extract dilution for the substrate to remove the matrix effects.

#### 3.4.6. In Vitro Test on Living Cells

##### Cell Culture and Proliferation Assay

The normal human keratinocyte cell line HaCaT (purchased from CLS cell lines service GmbH, now Cytion (https://www.cytion.com/HaCaT-Cells/300493, accessed on 25 April 2024)) and the cervical adenocarcinoma cell line HeLa (obtained from the American Type Culture Collection (ATCC No. CCL-2, https://www.atcc.org/products/ccl-2, accessed on 25 April 2024)) were cultured in Dulbecco’s Modified Eagle Medium (DMEM) with 10% (*v*/*v*) Fetal Bovine Serum (FBS) until they reached 80–90% confluence under standard conditions (5% CO_2_, humidified atmosphere, and 37 °C). A proliferation assay was conducted using the CellTiter 96^®^ AQueous One Solution Cell Proliferation assay (Promega, Madison, WI, USA) following the manufacturer’s instructions and the method previously described by Grillo et al. [22]. Lyophilized extracts were dissolved in water as follows and then sterile-filtered through 0.22 µm filters: MAE 1° (25 mg/mL), MASWE 2° (50 mg/mL), and Conv. (50 mg/mL). Cells were seeded in 96-well plates at a concentration of ∼3 × 10^4^ cells per well in 100 µL of medium. Twenty-four hours after seeding, HeLa and HaCaT cells were treated with the extracts that were further diluted in DMEM to the final tested volume ratios (0.5, 1.0, 2.5, and 5.00 (*v*/*v*)). The experiments were performed in triplicate with four parallels for each volume ratio and sample. The control cells were untreated cells. The cell viability was calculated as a percentage of treated cells relative to control cells and presented as the mean ± S.D. The differences between means were analyzed using the ANOVA test, followed by Tukey’s post hoc test. A significant difference was considered at a *p*-value of <0.05.

##### Measurement of Reactive Oxygen Species

Reactive oxygen species (ROS) formation was measured spectrofluorometrically using a DCF-DA assay as described by Logarusic et al. [67]. In brief, HeLa and HaCaT cells were seeded in 96-well black plates at a concentration of 3 × 10^4^ cells/mL and incubated for 24 h. The following day, the cells were pretreated with MAE 1°, MASWE 2°, and Conv. (1.0 *v*/*v*) for 20 h. Cellular oxidation was then induced by adding 100 μM of H_2_O_2_ for 3 h. Afterward, the cells were washed with PBS, and 100 μL of 2′,7′-dichlorofluorescin-diacetate (DCF-DA) solution (50 μM) was added to each well. After a 30 min incubation period in the dark at 37 °C, fluorescence was measured using a Varian Cary Eclipse fluorescence spectrophotometer (Varian, Palo Alto, CA, USA) at a certain excitation wavelength (λex = 485 ± 10 nm) and emission wavelength (λem = 530 ± 12 nm). The experiments were performed in duplicate with four parallels for each sample. The differences between means were analyzed using the ANOVA test, followed by Tukey’s post hoc test. A significant difference was considered at a *p*-value of <0.05

#### 3.4.7. Thermal Stability

The MASWE optimized extract (MASWE 2°, 400 mg) was combined with the MAE optimized extract (MAE 1°, 600 mg) in a 2:3 ratio in 50 mL of solvent and mixed for 2 h at 500 rpm prior to freeze-drying. The resulting formulation, along with the optimal MAE extract, a control sample, and a conventional extract, underwent thermal stability testing. Each extract was dissolved in distilled water, and anthocyanin degradation was measured after incubation at four temperatures (45 °C, 60 °C, 80 °C, and 100 °C), resulting in eight samples per temperature. Degradation kinetics were evaluated by comparing different linearization models, with time plotted on the x-axis and the anthocyanin concentration expressed as M, ln(M), or 1/M on the y-axis. The ln(M) linearization, indicative of a first-order reaction, was selected as the best fitting model. Degradation constants (k values) were determined as the negative slopes of linear regression lines for each temperature. Ln(k) values were then plotted against the reciprocal of the absolute temperature (1/T) to extrapolate the activation energy (AE) and pre-exponential factor (A) using the Arrhenius equation.

#### 3.4.8. A Sustainability Assessment of the MAE Process

The sustainability level of the different protocols (MAE 1°, MASWE 2°, the combined approach, and the conventional benchmark) were assessed using various green metrics (see Equations (6)–(9)). Firstly, the most common metrics applied in green chemistry were tested as the Reaction Mass Efficiency (RME), E-Factor, and Process Mass Intensity and Efficiency (PMI and PME) [31].

(6) $$Reaction\;Mass\;Efficiency\;\left(RME,\;\%\right)=\frac{Mass\;of\;Product}{Mass\;of\;Matrix\;}\times 100$$

(7) $$E-Factor=\frac{Total\;Mass\;of\;Waste}{Mass\;of\;Product\;}$$

(8) $$Process\;Mass\;Intensity\;\left(PMI\right)=\frac{Total\;Mass\;in\;the\;Process}{Mass\;of\;Product\;}$$

(9) $$Process\;Mass\;Efficiency\;\left(PME,\;\%\right)=\frac{1}{PMI}\times 100$$

Subsequently, more complex green metrics that account for energy consumption and the use of hazardous solvents were tested and compared. The metrics, including the Green Certificate, AgreePrep, ComplexGapi, and Green Motion, were considered to provide a comprehensive sustainability assessment of the procedures [32,33,34,35].

The Green Motion metric developed by Mane is a quantitative tool for assessing the environmental impact of chemical processes based on the 12 principles of Green Chemistry. It assigns a score from 0 to 100, with a higher score indicating a safer and more eco- friendly process. The metric evaluates aspects such as the origin of raw materials, the use of solvents, reaction efficiency, and waste production, providing a comprehensive overview of health, safety, and environmental factors. This versatile tool can be used from lab-scale research to industrial production.

## 4. Conclusions

This study highlights the potential of BP as a valuable source of bioactive compounds, utilizing green extraction technologies to recover anthocyanins and other polyphenols. Our results demonstrate that the extracts obtained by MAE and MASWE exhibit significant bioactivity, including antioxidant, anti-inflammatory, and antiproliferative properties.

The optimized extraction protocols not only ensure a high yield of bioactive compounds but also comply with green metrics and sustainability principles. Degradation studies revealed that the stability of anthocyanins is significantly enhanced when combined with MASWE extracts, which are rich in strongly antioxidant polyphenols. This synergistic approach preserves the integrity of the bioactive compounds, enhancing the thermal stability of the formulation.

Future research should focus on scaling up these green extraction processes from lab-scale to industrial levels, assessing their economic viability and further exploring the stability and bioactivity of the extracts in various product formulations. Additionally, deepening the investigation on the fractionation/isolation of active molecules could open new avenues in the development of cost-effective and sustainable pharmaceuticals. Furthermore, the use of more omni-comprehensive sustainability measurements such as the Life Cycle Assessment is still lacking and will provide a great contribution for assessing the advantages of green extraction techniques.

In summary, this study provides a robust framework for the valorization of agri-food by-products, contributing to sustainable development and the advancement of natural bioactive products. The integration of green extraction technologies and the circular economy model can drive innovation, promoting the development of effective and eco-friendly production.

## Figures and Tables

**Table 1:** BP extracts’ enzyme inhibition activity potential. Inhibition activity reported as EC50 of relative standard inhibitor equivalent: (i) α-amylase in acarbose equivalent (AE); (ii) β-glucosidase in castanospermine equivalent (CE); (iii) acetylcholinesterase in donepezil equivalent (DE); (iv) tyrosinase in kojic acid equivalent (KAE).

	α-Amylase mmol_AE_/g_extract_	β-Glucosidase mmol_CE_/g_extract_	Acetylcholinesterase µmol_DE_/g_extract_	Tyrosinase mmol_KAE_/g_extract_
MAE 1°	0.021 (0.015 ÷ 0.030)	0.017 (0.013 ÷ 0.021)	0.044 (0.036 ÷ 0.052)	0.118 (0.109 ÷ 0.127)
MASWE 2°	ND *	0.010 (0.008 ÷ 0.013)	0.076 (0.059 ÷ 0.098)	0.607 (0.515 ÷ 0.710)
Conv.	ND *	0.0167 (0.021 ÷ 0.013)	0.039 (0.024 ÷ 0.061)	ND *

* ND: EC50 not detectable.

## Data Availability

Data are contained within the article.

## Appendix A

**Table A1:** Dry yield, total phenolic content, and anthocyanin selectivity of BPs extracts compared to hydroalcoholic benchmark.

	Dry Yield	TPC	TAC
	mg_extract_/g_matrix_	mg_GAE_/g_extract_	mg_Cya-3-glu_/g_extract_
**MAE 1°**	94.94	109.93	22.23
**MASWE 2°**	47.47	151.67	0
**Conv.**	100.51	233.43	35.92

**Table A2:** Kinetic reaction order equation applied for evaluation of thermal anthocyanin degradation reaction.

Reaction Order	Differential Rate Law	Integrated Rate Law	Characteristic Kinetic Plot	Slop of Kinetic Plot	Units of Rate Constant
Zero	$\frac{-d\;[A]}{d\;t}=k$	$\left[A\right]={\left[A\right]}_{0}-kt$	$\left[A\right]\;vs.\;t$	−k	mole L^−1^ s^−1^
First	$\frac{-d\;[A]}{d\;t}=k\left[A\right]$	$\left[A\right]={\left[A\right]}_{0}\;e^{-kt}$	$\ln\left[A\right]\;vs.\;t$	−k	s^−1^
Second	$\frac{-d\;[A]}{d\;t}=k{\left[A\right]}^{2}$	$\left[A\right]=\frac{{\left[A\right]}_{0}}{1+kt\;{\left[A\right]}_{0}}$	1/[A]vs. t	k	L mole^−1^ s^−1^

**Table A3:** The kinetic reaction orders considered for the thermal anthocyanin degradation reaction. The three possible orders were evaluated by comparing the R^2^ values obtained at different temperatures.

MAE 1°			
	Reaction Order Zero [M] vs. time	First-Order Reaction ln[M] vs. time	Second-Order Reaction 1/[M] vs. time
45 °C	y = −0.00001x + 0.1417 R^2^ = 0.9345	y = −0.0002x − 1.8975 R^2^ = 0.9963	y = 0.0022x + 5.3264 R^2^ = 0.9634
60 °C	y = −0.000077x + 0.181890 R^2^ = 0.9765	y = −0.0006x − 1.673 R^2^ = 0.9934	y = 0.0054x + 4.9881 R^2^ = 0.9856
80 °C	y = −0.000293x + 0.172064 R^2^ = 0.9373	y = −0.0029x − 1.7104 R^2^ = 0.9562	y = 0.033171x + 4.749454 R^2^ = 0.9300
100 °C	y = −0.000700x + 0.154423 R^2^ = 0.8392	y = −0.012738x − 1.712313 R^2^ = 0.9854	y = 0.482027x − 5.405676 R^2^ = 0.8582
**Contr.**			
	**Reaction Order Zero** **[M] vs. time**	**First-Order Reaction** **ln[M] vs. time**	**Second-Order Reaction** **1/[M] vs. time**
45 °C	y = −0.000015x + 0.169502 R^2^ = 0.9367	y = −0.000169x − 1.702361 R^2^ = 0.9967	y = 0.00234x + 3.82670 R^2^ = 0.9271
60 °C	y = −0.0001x + 0.2279 R^2^ = 0.9622	y = −0.000856x − 1.425694 R^2^ = 0.9933	y = 0.00696x + 3.57196 R^2^ = 0.9748
80 °C	y = −0.0004x + 0.2052 R^2^ = 0.9015	y = −0.004018x − 1.510549 R^2^ = 0.9765	y = 0.04891x + 3.03949 R^2^ = 0.9749
100 °C	y = −0.0009x + 0.1986 R^2^ = 0.8397	y = −0.012895x − 1.469994 R^2^ = 0.9964	y = 0.36898x − 3.73888 R^2^ = 0.8827
**Conv.**			
	**Reaction Order Zero** **[M] vs. time**	**First-Order Reaction** **ln[M] vs. time**	**Second-Order Reaction** **1/[M] vs. time**
45 °C	y = −0.000007x + 0.100726 R^2^ = 0.9234	y = −0.000111x − 2.266225 R^2^ = 0.9819	y = 0.00185x + 8.88767 R^2^ = 0.9838
60 °C	y = −0.000046x + 0.114338 R^2^ = 0.9052	y = −0.000565x − 2.150258 R^2^ = 0.9466	y = 0.00719x + 8.27849 R^2^ = 0.9696
80 °C	y = −0.0002x + 0.1039 R^2^ = 0.8765	y = −0.003116x − 2.226536 R^2^ = 0.9540	y = 0.05973x + 8.00636 R^2^ = 0.9841
100 °C	y = −0.0005x + 0.0977 R^2^ = 0.7721	y = −0.012295x − 2.228502 R^2^ = 0.9842	y = 0.61166x − 2.37868 R^2^ = 0.9304
**Form. 1° + 2°**			
	**Reaction Order Zero** **[M] vs. time**	**First-Order Reaction** **ln[M] vs. time**	**Second-Order Reaction** **1/[M] vs. time**
45 °C	y = −0.000008x + 0.094515 R^2^ = 0.9381	y = −0.000157x − 2.298634 R^2^ = 0.9932	y = 0.00356x + 7.78990 R^2^ = 0.9641
60 °C	y = −0.000056x + 0.115428 R^2^ = 0.9778	y = −0.000775x − 2.111502 R^2^ = 0.9949	y = 0.01180x + 7.30491 R^2^ = 0.9705
80 °C	y = −0.0002x + 0.1072 R^2^ = 0.9255	y = −0.003301x − 2.167922 R^2^ = 0.9880	y = 0.06732x + 6.55359 R^2^ = 0.9431
100 °C	y = −0.0005x + 0.0996 R^2^ = 0.7707	y = −0.010892x − 2.262965 R^2^ = 0.9227	y = 0.39325x + 5.05235 R^2^ = 0.9807

**Table A4:** The investigated parameters of the different green metrics. * The term “Percentage of applicable criteria (%)” refers to the level of transposition of the criteria applied for green analytics in green extraction techniques for the relative green metric.
